# Comparative Pathway Integrator: A Framework of Meta-Analytic Integration of Multiple Transcriptomic Studies for Consensual and Differential Pathway Analysis

**DOI:** 10.3390/genes11060696

**Published:** 2020-06-24

**Authors:** Xiangrui Zeng, Wei Zong, Chien-Wei Lin, Zhou Fang, Tianzhou Ma, David A. Lewis, John F. Enwright, George C. Tseng

**Affiliations:** 1 Computational Biology Department, Carnegie Mellon University, Pittsburgh, PA 15213, USA; xiangruz@andrew.cmu.edu; 2 Department of Biostatistics, University of Pittsburgh, Pittsburgh, PA 15260, USA; WEZ97@pitt.edu (W.Z.); fangz.ark@gmail.com (Z.F.); 3 Division of Biostatistics, Medical College of Wisconsin, Wauwatosa, WI 53226, USA; chlin@mcw.edu; 4 Department of Epidemiology and Biostatistics, University of Maryland, College Park, MD 20742, USA; tma0929@umd.edu; 5 Department of Psychiatry, University of Pittsburgh, Pittsburgh, PA 15260, USA; lewisda@upmc.edu (D.A.L.); enwrightjf@upmc.edu (J.F.E.)

**Keywords:** pathway, meta-analysis, text mining

## Abstract

Pathway enrichment analysis provides a knowledge-driven approach to interpret differentially expressed genes associated with disease status. Many tools have been developed to analyze a single study. However, when multiple studies of different conditions are jointly analyzed, novel integrative tools are needed. In addition, pathway redundancy introduced by combining multiple public pathway databases hinders interpretation and knowledge discovery. We present a meta-analytic integration tool, Comparative Pathway Integrator (CPI), to address these issues using adaptively weighted Fisher’s method to discover consensual and differential enrichment patterns, a tight clustering algorithm to reduce pathway redundancy, and a text mining algorithm to assist interpretation of the pathway clusters. We applied CPI to jointly analyze six psychiatric disorder transcriptomic studies to demonstrate its effectiveness, and found functions confirmed by previous biological studies as well as novel enrichment patterns. CPI’s R package is accessible online on Github metaOmics/MetaPath.

## 1. Introduction

In a typical transcriptomic study, a set of candidate genes associated with diseases or other outcomes are first identified through differential expression analysis. Then, to gain more insight into the underlying biological mechanism, pathway analysis (also known as gene set analysis) is usually applied to pursue functional annotation of the candidate biomarker list. The goal behind pathway analysis is to determine whether the detected biomarkers are enriched in pre-defined biological functional domains. These functional domains might come from one of the publicly available databases such as GO [1], Reactome [2] and KEGG [3], or one of the integrated pathway collection such as MSigDB [4] and Pathway Commons [5]. Three main categories of pathway analysis methods have been developed in the past decade. The first category of methods called “over-representation analysis" considers biomarkers under a certain cutoff of differential express (DE) evidence and statistically evaluates the fraction of DE genes in a particular pathway found among the background genes. Without a hard threshold, the second category “functional class scoring" takes the DE evidence scores of all genes in a pathway into account and aggregates them into a single pathway-specific statistics. The third category “pathway topology" further incorporates the information of gene-gene interaction and their cellular location in addition to the pathway database. Details of general pathway enrichment analysis review can be found in [6].

Many transcriptomic datasets have been generated with the rapid advances of high-throughput experimental technologies in the past decade. Meta-analysis, a set of statistical methods for combining multiple studies of a related hypothesis, has thus become popular [7]. Some methods have been developed for the pathway meta-analysis. Shen and Tseng [8] developed two approaches of meta-analysis for pathway enrichment by combining DE evidence at the gene level (MAPE_G) or at the pathway level (MAPE_P). Nguyen et al. [9] proposed a robust bi-level pathway meta-analysis by adding an intra-experiment level analysis and another data-driven meta-analysis approach, DANUBE [10], using unbiased empirical distribution. However, in many real applications, when multiple datasets for a common biological hypothesis are available but possibly performed under different conditions (e.g., different tissues, different cell composition or different experimental platforms), it becomes necessary to detect both pathways enriched consistently in all studies (consensually enriched pathways) and pathways enriched in partial studies (differentially enriched pathways). One naïve way is to identify the enriched pathways in each study individually and manually check whether a certain pathway is enriched in one or multiple studies (e.g., using Venn diagram for enriched pathways in each study under a certain FDR threshold). This approach is sensitive to the choice of FDR threshold and is ad hoc in drawing a final conclusion. To avoid an arbitrary significance threshold, Plaisieret et al. [11] and Cahill et al. [12] have proposed rank-rank hypergeometric overlap (RRHO) plot to visualize contrasting enrichment significance under all continuous significance level of two studies. This approach is, however, limited to comparing two studies. To the best of our knowledge, there is currently no available statistical tool that can achieve the goal of integrating pathway enrichment of multiple studies in an automated and systematic manner for characterizing consensually and differentially enriched pathways.

A second issue emerges with pathway enrichment analysis is the pathway redundancy across pathway databases. Researchers often have difficult time to infer and interpret the underlying biological mechanism without presumed bias due to the large number of pathways identified. This kind of redundancy frequently occurs in a regular pathway enrichment analysis since different pathway databases contain similar annotated pathways with highly overlapped genes. The DAVID Bioinformatics Resources [13] partially resolved this issue by clustering pathways based on a kappa statistic representing the pathway similarity. However, the users still had to manually inspect each pathway in a cluster. Due to the long and vague descriptions in many pathways, users can still struggle to reach a solid conclusion from the results.

In light of the aforementioned drawbacks in existing tools, we propose a meta-analytic integrative framework to combine multiple transcriptomic studies for identifying consensual and differential pathway enrichment, wrapped in a tool named Comparative Pathway Integrator (CPI). CPI incorporates 25 pathway databases, including GO [1], Reactome [2], KEGG [3] and MSigDB [4] or user-defined gene set lists, as reference of pathway analysis. In order to identify both consensual and differential enriched pathways across studies, we applied the adaptively weighted Fisher’s method [14], which was originally developed to combine *p*-values from multiple omics studies for detecting homogeneous and heterogeneous differentially expressed genes. Next, cluster analysis based on pathway similarity (defined by gene overlap) is applied to remove the level of pathway redundancy. But unlike DAVID, we adopt a tight clustering algorithm similar to [15] to allow scattered pathways without being clustered and derive tight pathway clusters. Subsequently, we developed a text mining algorithm to automate the annotation of the tight pathway clusters by extracting keywords from pathway descriptions, which also offers more statistically valid summarization compared to leaving user to manually explore pathways in a cluster. Lastly, CPI provide users with both spreadsheets and graphical outputs for intuitive visualization, statistically solid presentation and insightful interpretation. CPI has a standalone R package as well as being disseminated into MetaOmics, an analysis pipeline and browser-based software suite for transcriptomic meta-analysis [16].

## 2. Materials and Methods

### 2.1. Workflow of Comparative Pathway Integrator (CPI)

**Figure 1:**
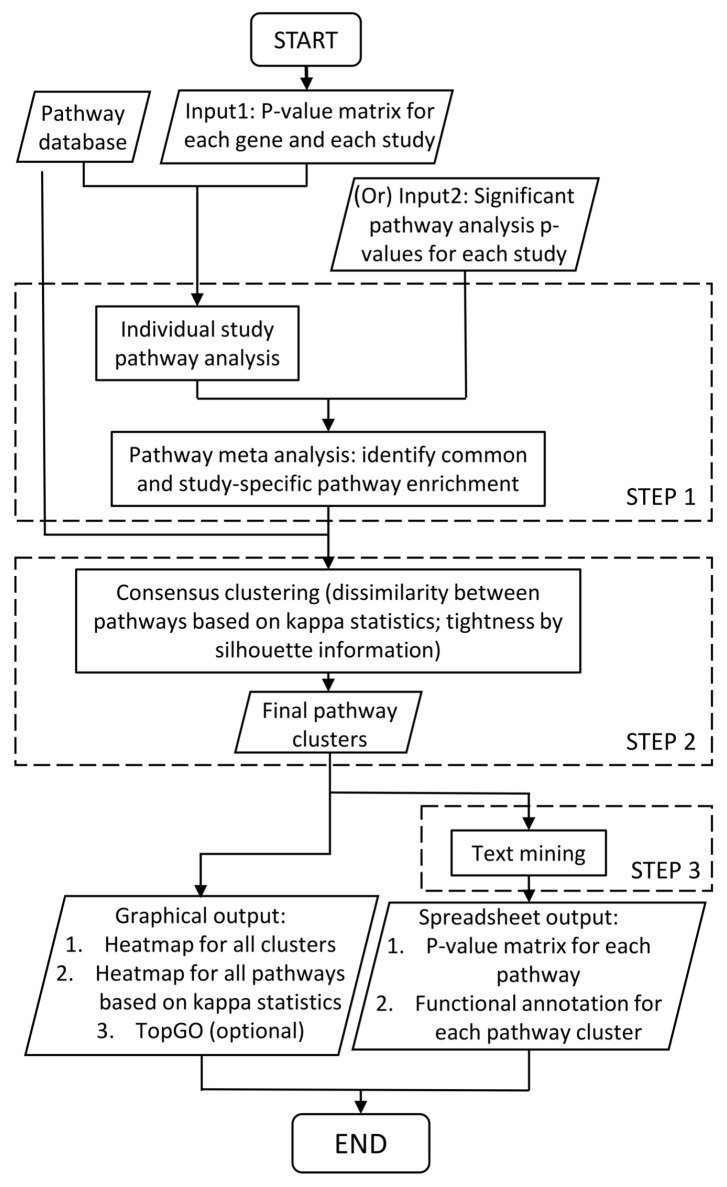
Workflow of Comparative Pathway Integrator (CPI).

CPI is a comprehensive tool incorporating several widely accepted mature methods as well as multiple novel algorithms/approaches. It is mainly composed of three steps (Figure 1). The first step (Section 2.2) performs meta-analytic pathway analysis, which integrates pathway enrichment analysis and meta-analysis. This step partially resembles the previous in-house work of R package MetaPath [8] but with advanced features. While MetaPath focuses on detecting consensually enriched pathways, CPI will detect both consensual and differentially enriched pathways, providing valuable information on how the patterns of pathway enrichment differ across studies. The second step (Section 2.3) implements pathway clustering. This step aims to reduce redundancy of the pathway information from commonly hundreds of enriched pathways to only handful (usually 5–10) pathway clusters. The results are more succinct and interpretable. The third step (Section 2.4) includes text mining based on pathway names and descriptions to find keywords characterizing the intrinsic biological functions of each pathway cluster. A permutation-based statistical test is performed to assess if a specific biological noun phrase appears significantly more than by chance. Without this step, it would be difficult to avoid subjective biases from users and to objectively identify the representative biological mechanisms for each pathway cluster since clustering of the pathways does not fundamentally reduce the total number of pathways under investigation. Finally, we generate graphical and spreadsheet outputs of pathway *p*-value matrices and pathway clustering details including gene composition and functional keywords.

### 2.2. Meta-Analytic Pathway Analysis

Compared to differential expression analysis, pathway enrichment analysis provides more biological insight in a more systematic and comprehensive manner. In CPI, we allow multiple methods of over representation analysis since recent comparative studies [17,18] have shown little additional advantages using sophisticated functional class scoring or pathway topology methods. But if such advanced pathway enrichment analysis is preferred, users can externally implement the pathway analysis and CPI can accept lists of significant pathways and the corresponding *p*-values as alternative input (Input 2 in Figure 1). Given pathway enrichment results, we perform adaptively-weighted Fisher’s (AW-Fisher) method [14,19] for meta-analysis, to identify pathways significant in one or more studies/conditions. AW-Fisher not only increases statistical power, but also provides a 0/1 binary weight for each study, indicating whether a study contributes to the meta-analytic significance. Given a user-specified q-value cutoff, we obtain a list of significant pathways, with 0/1 binary weights indicating whether a pathway is significantly enriched across most or all studies/conditions (i.e., consensually enriched pathways) or only in partial studies (i.e., differentially enriched pathways). For example, in Section 3, the “GO:MF kinase activity” pathway has raw enrichment *p*-values $\left(0.26922,0.17773,0.06485,2.04\times 10^{-5},0.00449,0.018922\right)$ for the six studies. AW-Fisher meta-analysis generates combined *p*-value = $5.52\times 10^{-6}$ (q-value = 0.00014) with adaptive weights = (0,0,1,1,1,1), showing enrichment in the last four bipolar studies or major depressive disorder studies but not in the first two schizophrenia studies.

### 2.3. Pathway Clustering for Reducing Redundancy and Enhancing Interpretation

Because of the nature of pathway definitions (e.g., hierarchy structure or overlapping functions), many genes are shared among different pathways. Similar pathways can also repeat in different pathway databases with slightly different gene composition, annotation or description. Such redundancies often stumble interpretation of pathway analysis results. In CPI, we perform pathway clustering to reduce the redundancy among detected pathways. The similarity between different pathways is calculated based on kappa statistics [20], which depends on how many genes are mutually identical or exclusive among those pathways. The kappa statistics represents the dissimilarity between two pathways based on the genes composing each pathway. Based on the dissimilarity matrix of all pathway pairs, consensus clustering [21] is used to estimate the number of clusters. Following the original consensus clustering method, an elbow plot and consensus CDF plot are generated to assist users to decide the number of clusters.

We next assign detected pathways into clusters. For most clustering algorithms including the aforementioned consensus clustering, all pathways are forced into clusters although it is well-known that leaving scattered subjects out of clusters often generate tighter clusters and improve the clustering performance in such high-dimensional data [15,22,23]. In CPI, we allow scattered pathways to form singletons, when its gene composition is largely different from representative pathway clusters, to avoid adding outliers to the pathway clusters. To improve the tightness of the clusters, we further calculated for each pathway the silhouette width [24], a measure of how tightly each pathway is grouped in its cluster, and removed the scattered pathways with low silhouette width iteratively until all pathways’ silhouette widths are above a certain cutoff. The removing cutoff for silhouette width is estimated empirically based on its distribution as in our multi-disease application in Section 3 (we choose 0.1 in this paper). For the identified singleton pathways, we collected them to form a scattered pathway set instead of filtering them out. In general applications, we recommend users to investigate the identified tight pathway clusters first with the subsequent text mining tool introduced in the next subsection since these pathway clusters are better annotated in the pathway databases and are likely better studied in the current biological knowledge domain. We, however, do not discard pathways in the scattered set but recommend them in the secondary investigation.

### 2.4. Text Mining for Automated Annotation and Knowledge Retrieval of Pathway Clusters

#### 2.4.1. Motivation and Problem Setting

Although the pathway cluster analysis in Section 2.3 can greatly reduce redundancy structure of the detected pathways, users still have to manually scan through all pathways in a cluster to grasp its major content and biologically driving mechanism, which can be labor-intensive and subject to the user’s biased presumption. Therefore, we need a more rigorous and statistically meaningful summary of the pathway cluster to guide an unbiased interpretation. The above goal is expressed here as the text mining for key noun phrases of each pathway cluster: which noun phrase appears more frequently in a certain pathway cluster than by chance in statistical sense? We will therefore treat these noun phrases as the potentially representative entities (mechanism) for the pathway cluster. The entity is counted based on the number of pathways containing it, rather than the frequency of it appearing in all pathway descriptions in a cluster. For instance, in a certain cluster, if "T cell" occurs six times in 3 pathway descriptions: three times in pathway #1, twice in pathway #2 and once in pathway #3, T cell" is counted 3 occurrences even though it appears 6 times in total.

#### 2.4.2. Pathway-Phrase Matrix

**Figure 2:**
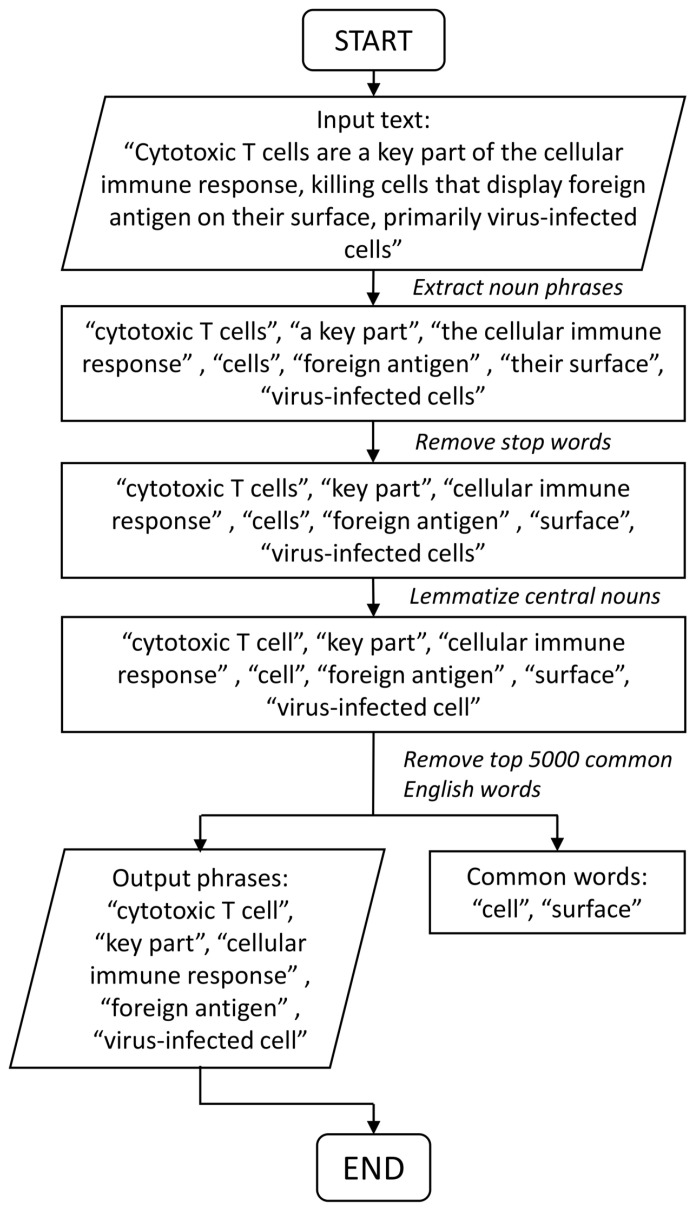
Workflow of noun phrase extraction.

For each pathway description, we firstly extracted unique noun phrases from it. This step was done using the spacy_extract _nounphrases function from R package *spacyr* [25] which is an R wrapper around the Python *spaCy* package [26]. *spaCy* is an industrial strength text-mining package employing a large library database as well as some machine learning algorithms to detect information from texts. The stop words in English, such as “the”, “a”, “that”, which are common and carry no important information, are removed from those noun phrases by using the English stop words database from R package *tm* [27]. After removing all stop words, the last word of each noun phrases, i.e., the central noun of a noun phrase, is lemmatized (converting plural form to singular form) by the lemmatize_words function in *textstem* [28]. The top 5000 common English words [29] were then filtered out from the result noun phrases of length one. A text mining process of an example sentence is shown in Figure 2.

In total, we provide 25 pathway databases (GO, KEGG, BioCarta, Reactome, Phenocarta, etc.) with 26,801 pathways in CPI for users to select in the analysis. The above preprocessing and filtering steps were repeated for each pathway to generate standard noun phrases for all pathways. Based on the results, we constructed a binary matrix where each row being a noun phrase and each column being a pathway with element $w_{ij}=1$ indicating the pathway description *j* contains the noun phrase *i* and 0 otherwise.

Once the matrix was constructed, R package *wordnet* [30] was used to identify synonyms from row names of the matrix (noun phrases). When a pair of synonyms are identified, the phrase with lower occurrence in all pathways are combined with the phrase with higher occurrence. Then the row of less occurred phrase was deleted. Since in later text mining of pathway clusters, a phrase needs to at least occur in two pathways to be considered, all rows of phrases which occurred only once in the 26,801 pathways were deleted. As a result, a matrix of 36,037 rows and 26,801 columns was constructed.

For later penalized permutation test, the above text mining matrix construction procedure was also applied to pathway names of 26,801 pathways, producing a similar matrix of 36,037 rows and 26,801 columns with $v_{ij}=1$ indicating the pathway name *j* contains the noun phrase *i* and 0 otherwise. For a given pathway (e.g., GO:0030964), the pathway name (“NADH dehydrogenase complex”) is usually more concise while the pathway description (“An integral membrane complex that possesses NADH oxidoreductase activity. The complex is one of the components of the electron transport chain. It catalyzes the transfer of a pair of electrons from NADH to a quinone”) gives a more detailed illustration of the pathway.

#### 2.4.3. Test Statistics for Noun Phrase Enrichment Analysis

A simple strategy to test for the significance of a phrase frequently appearing in a cluster is by simple counting and conducting Fisher exact test. Yet we found this method to be less powerful and less biologically justifiable from real data analysis, because the phrases in the term name or a shorter description of a pathway are deemed to be more representative than those in a full or longer description. In other words, phrases appearing in a pathway with shorter description should statistically contribute more weights than in a pathway with lengthy description because the latter is more likely to happen by chance. Therefore, we down-weighted the phrase count with long description by assigning a score between 0 and 1 to each pathway *j* to indicate whether it contains phrase *i*:

$$x_{ij}=\left\{\begin{array}{ll} 1 & v_{ij}=1\;(\mathrm{phrase}\;i\;\mathrm{appeared}\;\mathrm{in}\\ & \mathrm{the}j\text{-th}\;\mathrm{pathway}\;\mathrm{name}),\\ exp(-\alpha \cdot |w_{j}|) & v_{ij}=0\;\mathrm{and}\;w_{ij}=1\;(\mathrm{phrase}\\ & i\;\mathrm{appeared}\;\mathrm{only}\;\mathrm{in}\;\mathrm{the}\;j\text{-th}\\ & \mathrm{pathway}\;\mathrm{description}),\\ 0 & v_{ij}\;=\;w_{ij}\;=\;0 \end{array}\right.$$

where $|w_{j}|=\sum_{i}w_{ij}$ is the number of unique noun phrases in the description of pathway *j* and α is a parameter controlling the degree of penalty. The greater α is, the greater the penalty is on longer description. When α equals to 0, there is no penalty and our test simplifies to Fisher’s exact test with equal weight to pathway names and pathway descriptions. Based on evaluation in real data, α=0.05 is used in our package. Next, we define cluster score $T_{i}\left(C\right)$ to be the sum of scores of pathways in the cluster, i.e., for phrase *i* in a pathway cluster *C*, we define the test statistics:

$$T_{i}\left(C\right)=\sum_{j\in C}x_{ij}$$

#### 2.4.4. Permutation Test

To test for the null hypothesis that a phrase is not enriched in a certain cluster, we adopt a permutation analysis. For each phrase *i* in the *b*-th permutation, pathways are randomly sampled to form subset $S_{b}$ with the same cluster size as *C*. Test statistics $T_{i}\left(S_{b}\right)$ is recomputed at the end of each permutation. The operation is then repeated for a large number of times (say, B=10,000 times). Finally, all $T_{i}\left(S_{b}\right)$’s form a null distribution and are compared to the observed statistics $T_{i}\left(C\right)$. And the *p*-value could be calculated by $p\left(T_{i}\left(C\right)\right)=\frac{\sum_{b=1}^{B}I\left(T_{i}\left(C\right)\geq T_{i}\left(S_{b}\right)\right)}{B}$, indicating how extremely frequent phrase *i* is seen in cluster *C*. Multiple comparison is then corrected by Benjamini–Hochberg procedure [31] to control false discovery rate (FDR).

#### 2.4.5. Graphical and Spreadsheet Output

**Figure 3:**
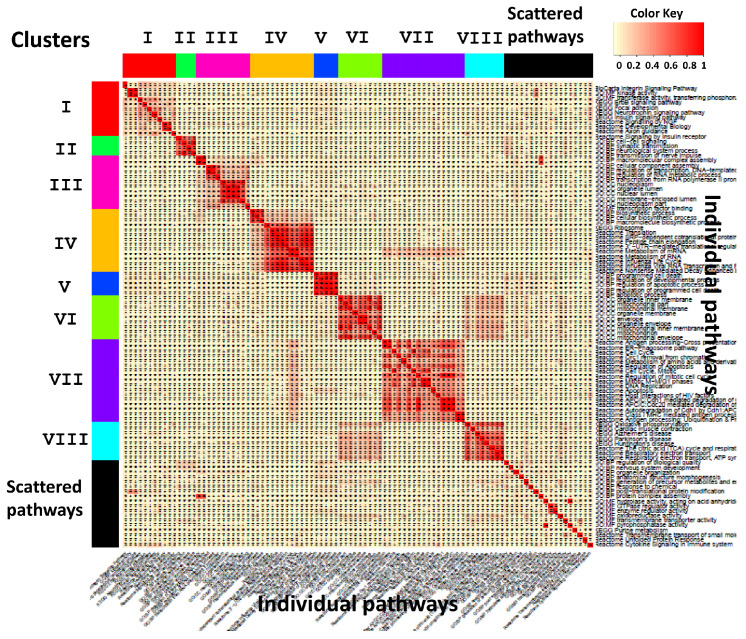
Heatmap of kappa statistics of pair-wise pathways in all clusters.

**Figure 4:**
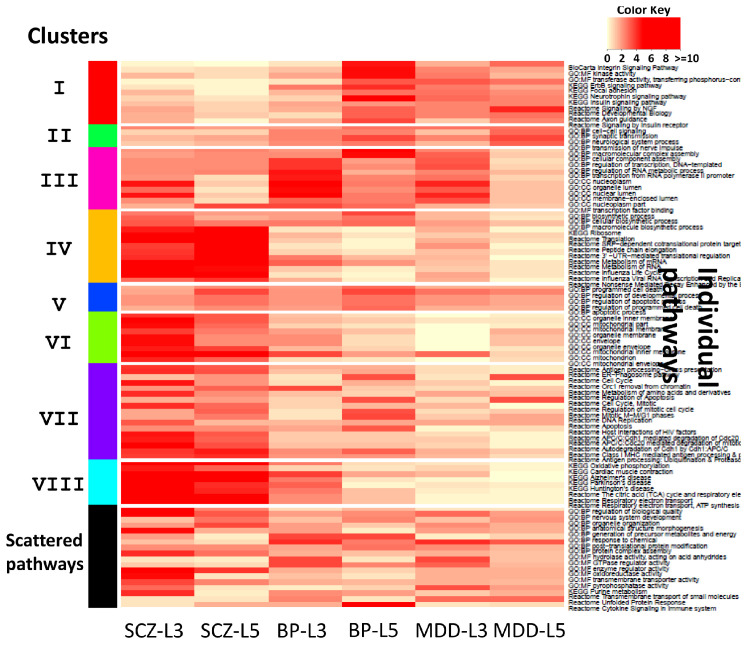
Heatmap of log10-scale pathway enrichment *p*-values of pathways annotated by eight pathway clusters (I-VIII) and a scattered pathway set (black).

**Figure 5:**
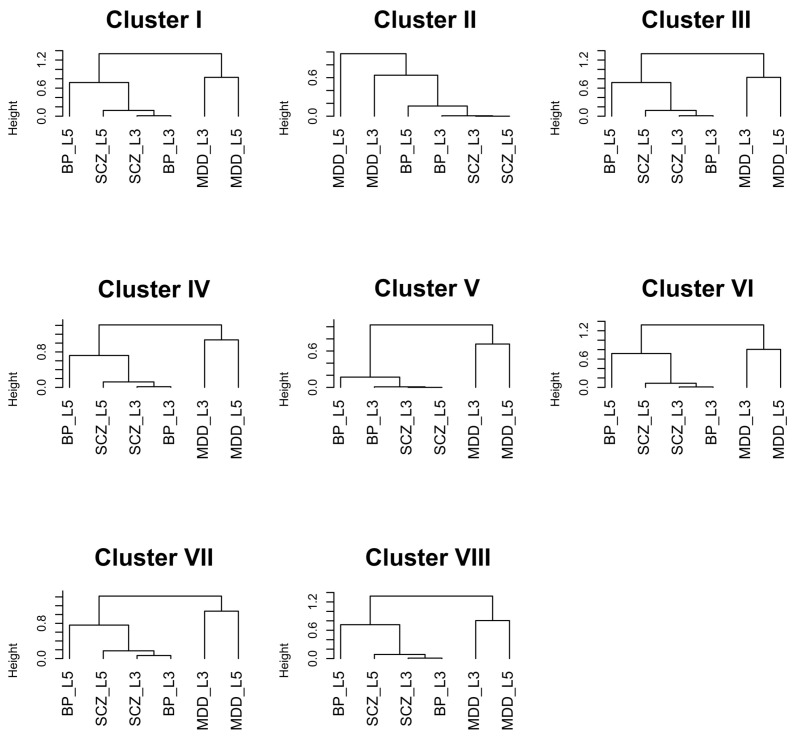
Hierarchical clustering of psychiatric studies in each cluster with distance defined by the log10-scale pathway enrichment *p*-values.

In the final step, CPI outputs visualization tools, including (1) heatmap of kappa statistics matrix for pair-wise pathways (see Figure 3) (2) heatmap of pathway enrichment *p*-value matrix (pathways sorted by clusters on the rows and studies on the columns) (see Figure 4) (3) multi-dimensional scaling (MDS) plot of pathways and cluster assignment distributed by kappa statistics (see Supplementary Figure S3c) and (4) dendrograms of hierachical clustering (distance measured by pathway enrichment *p*-values) of studies in each cluster (see Figure 5). CPI also provides diagnostic tools such as CDF plot and scree plot to determine the number of clusters in consensus clustering.

#### 2.4.6. Datasets and Databases

We provide 22 Homo sapiens pathway databases in CPI, including 14 pathway databases from MsigDB (containing GO, KEGG, Reactome, BioCarta, and others), 2 databases from Connectivity Map, transcription factor target database JASPAR, Protein-Protein interaction database and 3 microRNA target databases as options for enrichment analysis. In addition, GO and KEGG for Mus musculus and Saccharomyces cerevisiae, and JASPAR database for Mus musculus are also provided (see Supplementary Table S2). Users may choose to apply their own pathway databases with the extra computing cost of re-calculating the pathway-phrase matrix in Section 2.4.2.

## 3. Results

### 3.1. Application to Transcriptomic Data of Multiple Psychiatric Disorders

To demonstrate its utility, we applied CPI to an integrative analysis of a postmortem microarray dataset from three psychiatric disorders [32]. Briefly laser-microdissection was used to isolate pools of 100 pyramidal neurons from layers 3 (L3) and 5 (L5) from dorsolateral prefrontal cortex (DLPFC). Samples were collected from schizophrenia (SCZ), bipolar disorder (BP), and major depressive disorder (MDD) subjects and unaffected comparison subjects matched for age and sex. Samples consisted of both cell types from all subjects except for the L5 SCZ sample where two subjects were removed due to quality control issues. Following identification of differentially-expressed genes in each of the 6 diagnostic categories (cells from two layers and three different diagnoses) four default pathway databases in CPI (Gene Ontology, KEGG, Reactome and BioCarta) were used for this application.

Low expression and non-informative genes were first filtered out by quantile filtering and then differential expression (DE) analysis was conducted by limma embedded in the MAPE2.0 function from our package which allows both raw transcriptomic data input as well as DE *p*-value matrix provided by users. Pathway enrichment analysis was performed in each study using the top 400 DE genes and the results were meta analyzed by adaptively weighted Fisher’s method in CPI to obtain the final integrative *p*-values and q-values in each pathway. We filtered out pathways containing less than 15 genes or more than 500 genes in the pathway databases. Of the 1901 pathways analyzed, 96 pathways had meta-analyzed q-values smaller than 0.0005 and were entered for pathway cluster analysis. The number of pathway clusters were selected to be 8 which was justified by the elbow plot and consensus CDF plot (see Supplementary Figure S2a,b) and 18 pathways were left out as scattered pathways.

Figure 3 displays heatmap of kappa statistics of pair-wise pathways, sorted by the 8 identified pathway clusters and a scattered pathway set (black color). Figure 4 shows heatmap of log10-transformed pathway enrichment *p*-values with pathways on the rows and six studies on the columns. Dendrograms of hierachical clustering of studies in each cluster are shown in Figure 5. Table 1 contains 10 functionally annotated phrases identified from the penalized text mining algorithm for each pathway cluster. We note that, by our algorithm, heatmap pattern of pathways in the same cluster in Figure 4 may not visualize similarly since the pathway clusters are obtained by kappa statistics, representing similarity of gene content of any pair of pathways, rather than pathway enrichment *p*-values. But in general, we do observe clear pattern in almost all 8 clusters. For example, cluster VI, VII and VIII contain highly enriched pathways in SCZ-L3 and SCZ-L5, marginal enrichment in BP-L3 and BP-L5 but almost no significance in MDD-L3 and MDD-L5. Based on text mining results, clusters VI and VIII contain pathways related to *mitochondrion, ATP synthesis, NAD*, etc. Our results also suggest these alternations across DLPFC layer 3 and layer 5 are mainly related to ATP production rather than other aspects of mitochondrial function. Cluster VII with keywords *degradation, multiubiquitination, ubiquitin 26s proteasome system,* etc. is significantly altered in schizophrenia DLPFC layers and to a lesser extent in bipolar disorder. Similar results have been reported in a blood-based microarray investigation of both schizophrenia and bipolar disorder [33].

The result of cluster VI, VII and VIII is consistent to several biological findings in the literature. Firstly, our results are highly consistent with the original publication in [32]. Secondly, the paper [34] analyzed a mostly non-overlapping schizophrenia cohort and also showed that the differential expression genes at the layer 3 and/or layer 5 pyramidal cells in the DLPFC of schizophrenia subjects are mainly related to mitochondrial (MT) and ubiquitin-proteasome system (UPS) functions. The findings were followed up with qPCR validation in selected target genes. This is again consistent to our findings in cluster VI, VII and VIII. Finally, it has been shown that the synaptic area is particularly sensitive to MT and UPS deficits due to the high demand for ATP and for UPS activity at pre- and post- synaptic terminals [35], which is consistent with our results suggesting ATP production as the main aspect of the mitochondrial dysfunction in schizophrenia diseases.

Cluster I and cluster IV had a different pattern of pathway alterations. Cluster IV with keywords *metabolism, mRNA, ribosome, viral protein, NUMB,* etc. is significantly altered only in schizophrenia DLPFC layers, which indicates protein synthesis dysfunction. However, similar altered expression of gene sets related to protein synthesis has been found in postmortem hippocampus and orbitofrontal cortex of patients with major depression, bipolar disorder, and schizophrenia consensually [36]. This implies different degrees of protein synthesis pathway alterations in different brain tissues. Cluster I with keywords *insulin, NGF, BDNF, neurotrophins, Trk tyrosine kinase receptor,* etc. shows an enrichment pattern mainly in MDD and BP-L5 with little enrichment in SCZ of BP-L3. This suggests some similarity between the expression of these gene in layer 5 between BP and MDD subjects.

Pathways in cluster III are enriched in layer 3 of all three diseases (SCZ-L3, BP-L3 and MDD-L3) but to a lesser extent in layer 5 (SCZ-L5, BPL5 and MDD-L5). This cluster is annotated with keywords such as *transcription, nucleoplasm, chromosome, nuclear content, nucleolus, RNA*, indicating alterations in general aspects of nuclear function. Finally, cluster II and V with moderate enrichment in all six studies contain general neural disease related pathways with keywords such as *neuron, cell death and apoptotic process* and likely represent processes involved in neuronal survival.

Interestingly hierarchical clustering of the six categories of samples (cells from two layers and three different diagnoses) for each of the eight identified pathway clusters shows a similar pattern. Specifically, each of the 8 pathway clusters are most tightly clustered by diagnosis across layers suggesting similar alterations in layers 3 and 5 within a disease. Furthermore, across diagnoses BP and SCZ cluster more strongly with one another than with MDD. This is consistent with transcriptome [37,38,39] and genomic [40] findings suggesting similarities between SCZ and BP.

### 3.2. Justification to Penalize Pathway Description by Length in Text Mining

In pathway databases, some pathways come with only pathway names (usually less than 15 words) and some contains both pathway names and pathway descriptions (can be up to 1500 words). In Fisher’s exact test by simple count, occurrences of a noun phrase are treated the same when appearing in these two extreme cases. In this case, signals of important mechanism terms in pathway names can be masked due to its frequent occurrence in pathway descriptions, while some non-informative terms can be falsely detected from long pathway descriptions. The penalization by pathway description length in Section 2.4.3 helps improve the sensitivity and reduce false positives. For example, in the real application in Section 3, the noun phrase *apoptosis* in cluster 7 was ranked low in the Fisher’s exact test (r = 37, *p* = 0.051) while prioritized in permutation analysis of the penalized test statistics (r = 4, *p* < $10^{-4}$) (see Supplementary Table S1). Some meaningless words such as et al in cluster 7 was ranked high in Fisher’s exact test (r=11, *p* = $5.08\times 10^{-7}$), but ranked low by penalized statistics (r = 27 and *p* = 0.005). In our experience, B=10,000 permutations are sufficient to obtain accurate *p*-value assessment while under a reasonable computing time. The entire permutation analysis for the example in Section 3 required only 1.5 min under parallel computing using ten cores.

## 4. Discussion

CPI has three advantages compared to existing methods. Firstly, CPI explores consensual and differential pathway enrichment pattern simultaneously when combining multiple related studies. To our knowledge, CPI is the first method for this purpose. Secondly, CPI clusters pathways by gene composition similarity (i.e., kappa statistics) to reduce pathway redundancy. Finally, CPI uses a statistically evaluated text mining method to annotate mechanisms of each pathway cluster automatically without subjective human interpretation. In addition, the proposed penalized text mining algorithm by permutation test was shown to outperform conventional Fisher’s exact test in text mining. We applied the tool to six transcriptomic datasets spanning on three psychiatric disorders (SCZ, BP and MDD) and two layers in DLPFC (L3 and L5). The result identified multiple pathway clusters with enrichment patterns consistent with previous findings, such as mitochondrial ATP dysfunction in schizophrenia DLPFC layers, as well as other new findings.

The current CPI package has several limitations. Firstly, for single study pathway analysis, our tool currently only provides Fisher’s exact test and Kolmogorov–Smirnov test. Users, however, can externally apply advanced methods such as GSEA [41] or others and take the result as alternative input. Secondly, our text mining algorithm relies on the descriptions provided by pathway databases. For pathway databases without detailed descriptions (e.g., in some KEGG pathways), text mining algorithm cannot annotate them well. Thirdly, computation time is not ignorable, especially in the text mining step. To incorporate more studies or more pathway databases, scalable computing algorithms will be needed.

## 5. Conclusions

In this article, we developed an integrative framework for combining and comparing pathway analyses from multiple transcriptomic studies, namely Comparative Pathway Integrator (CPI). CPI performs meta-analytic pathway analysis, reduces pathway redundancy to condense knowledge discovered from the results and conducts text mining to provide statistically solid inference on interpreting results. CPI has three major steps. In the first step, users can input either gene-based differential expression *p*-value matrix or pathway-based enrichment *p*-value matrix for each study to start with. If *p*-values of genes are entered, pathway enrichment analysis is applied first within each study. Enriched pathways are passed down to meta-analysis where AW-Fisher is applied to discover consensually and differentially enriched pathways. In the second step, significant pathways from AW-Fisher meta-analysis are clustered using consensus clustering, with consensus CDF plot and elbow plot to assist users to choose the number of clusters [21]. Silhouette information is used to achieve cluster tightness by removing scattered pathways without being clustered. In the third step, a penalized text mining algorithm is used to annotate each pathway cluster for an unbiased knowledge learning from the experimental data and pathway database. With the penalized matrix provided in package, CPI requires 2.8 min under parallel computing using ten cores to integrate six studies and 9888 genes in the psychiatric example with 1901 pathways in default pathway databases and B = 10,000 in permutation analysis. An R package is available at Github metaOmics/MetaPath.

In summary, CPI is a meta-analytic tool for integrating multiple related transcriptomic studies in pathway enrichment analysis. As more and more transcriptomic datasets accumulate in the public domain, the need of such integrative analysis will become more and more prevalent. CPI can fill the gap and provide biological insight in such comparative and integrative tasks. In addition to transcriptomic studies, the framework is readily extensible to integrate pathway analysis of multiple related proteomic studies or multiple metabolomics studies.

## Figures and Tables

**Table 1:** Ten significant keywords with q-value < 0.05 in each pathway cluster.

Cluster	Keywords
I	insulin, NGF, focal adhesion, BDNF, neurotrophins, Trk tyrosine kinase receptor, insulin receptor substrate, insulin receptor tyrosine kinase, Ras MAPK pathway, FAK
II	neuron
III	transcription, nucleoplasm, chromosome, nuclear content, nucleolus, RNA
IV	metabolism, mRNA, ribosome, replication, chemical reaction, cRNA, vRNA, viral protein, NUMB, nucleus
V	cell death, apoptotic process, activation, endogenous cellular process, programmed cell death, apoptosis
VI	mitochondrion, organelle, mitochondrial envelope, organelle envelope, lipid bilayer, inner elumen facing lipid bilayer, semiautonomous self replicating organelle, tissue respiration, virtually eukaryotic cell, cytoplasm
VII	degradation, APC/C, apoptosis, CDC20, CDH1, mitotic protein, MHC, multiubiquitination, ubiquitin 26s proteasome system, exogenous antigen
VIII	respiratory electron transport, ATP synthesis, inner mitochondrial membrane, chemiosmotic gradient, brown fat, rotenone, FAD, mitochondrial matrix, body temperature, NAD

**Abbreviation**. NGF: *Nerve growth factor*, BDNF: *Brain-derived neurotrophic factor*, MAPK: *Mitogen-activated protein kinase*, FAK: *Focal adhesion kinase*, RNA: *Ribonucleic acid*, APC/C: *Anaphase-promoting complex*, MHC: *Major histocompatibility complex*, ATP: *Adenosine triphosphate*, FAD: *Flavin adenine dinucleotide*, NAD: *Nicotinamide adenine dinucleotide*, NUMB, CDC20, CDH1: *Gene names*.

## Supplementary Materials

The following are available online at https://www.mdpi.com/2073-4425/11/6/696/s1, Figure S1: *p*-values and q-values from individual pathway analysis and meta-analysis and AW-fisher weight for each pathway in each study, Figure S2: CDF plot of consensus clustering and delta plot of consensus clustering, Figure S3: Heatmap for Kappa statistic of pair-wise pathways and heatmap of log10-scale pathway enrichment *p*-values of pathways and MDS plot of pathways and cluster assignment distributed by kappa statistics and Hierarchical clustering of psychiatric studies in each cluster, Table S1: Fisher-exact test 2 × 2 table for “apoptosis” in cluster 7, Table S2: 22 Homo sapiens pathway database including 14 pathway database from MsigDB, 2 databases from Connectivity map, transcription factor target database JASPAR, Protein-Protein interaction database, 3 microRNA databases and 1 phenotype database as options for enrichment analysis.

## References

[1] Ashburner M., Ball C.A., Blake J.A., Botstein D., Butler H., Cherry J.M., Davis A.P., Dolinski K., Dwight S.S., Eppig J.T. (2000). Gene Ontology: Tool for the unification of biology. Nat. Genet..

[2] Fabregat A., Sidiropoulos K., Garapati P., Gillespie M., Hausmann K., Haw R., Jassal B., Jupe S., Korninger F., McKay S. (2015). The reactome pathway knowledgebase. Nucleic Acids Res..

[3] Kanehisa M., Goto S. (2000). KEGG: Kyoto encyclopedia of genes and genomes. Nucleic Acids Res..

[4] Liberzon A., Birger C., Thorvaldsdóttir H., Ghandi M., Mesirov J.P., Tamayo P. (2015). The molecular signatures database hallmark gene set collection. Cell Syst..

[5] Rodchenkov I., Babur O., Luna A., Aksoy B.A., Wong J.V., Fong D., Franz M., Siper M.C., Cheung M., Wrana M. (2020). Pathway Commons 2019 Update: Integration, analysis and exploration of pathway data. Nucleic Acids Res..

[6] Khatri P., Sirota M., Butte A.J. (2012). Ten years of pathway analysis: Current approaches and outstanding challenges. PLoS Comput. Biol..

[7] Tseng G.C., Ghosh D., Feingold E. (2012). Comprehensive literature review and statistical considerations for microarray meta-analysis. Nucleic Acids Res..

[8] Shen K., Tseng G.C. (2010). Meta-analysis for pathway enrichment analysis when combining multiple genomic studies. Bioinformatics.

[9] Nguyen T., Tagett R., Donato M., Mitrea C., Draghici S. (2016). A novel bi-level meta-analysis approach: Applied to biological pathway analysis. Bioinformatics.

[10] Nguyen T., Mitrea C., Tagett R., Draghici S. (2016). DANUBE: Data-driven meta-ANalysis using UnBiased empirical distributions—Applied to biological pathway analysis. Proc. IEEE.

[11] Plaisier S.B., Taschereau R., Wong J.A., Graeber T.G. (2010). Rank–rank hypergeometric overlap: Identification of statistically significant overlap between gene-expression signatures. Nucleic Acids Res..

[12] Cahill K.M., Huo Z., Tseng G.C., Logan R.W., Seney M.L. (2018). Improved identification of concordant and discordant gene expression signatures using an updated rank-rank hypergeometric overlap approach. Sci. Rep..

[13] Huang D.W., Sherman B.T., Tan Q., Kir J., Liu D., Bryant D., Guo Y., Stephens R., Baseler M.W., Lane H.C. (2007). DAVID Bioinformatics Resources: Expanded annotation database and novel algorithms to better extract biology from large gene lists. Nucleic Acids Res..

[14] Li J., Tseng G.C. (2011). An adaptively weighted statistic for detecting differential gene expression when combining multiple transcriptomic studies. Ann. Appl. Stat..

[15] Tseng G.C., Wong W.H. (2005). Tight clustering: A resampling-based approach for identifying stable and tight patterns in data. Biometrics.

[16] Ma T., Huo Z., Kuo A., Zhu L., Fang Z., Zeng X., Lin C.W., Liu S., Wang L., Liu P. (2019). MetaOmics: Analysis pipeline and browser-based software suite for transcriptomic meta-analysis. Bioinformatics.

[17] Tarca A.L., Bhatti G., Romero R. (2013). A comparison of gene set analysis methods in terms of sensitivity, prioritization and specificity. PLoS ONE.

[18] Bayerlová M., Jung K., Kramer F., Klemm F., Bleckmann A., Beißbarth T. (2015). Comparative study on gene set and pathway topology-based enrichment methods. BMC Bioinform..

[19] Huo Z., Tang S., Park Y., Tseng G. (2019). *p*-value evaluation, variability index and biomarker categorization for adaptively weighted Fisher’s meta-analysis method in omics applications. Bioinformatics.

[20] Viera A.J., Garrett J.M. (2005). Understanding interobserver agreement: The kappa statistic. Fam. Med..

[21] Monti S., Tamayo P., Mesirov J., Golub T. (2003). Consensus clustering: A resampling-based method for class discovery and visualization of gene expression microarray data. Mach. Learn..

[22] Maitra R., Ramler I.P. (2009). Clustering in the Presence of Scatter. Biometrics.

[23] Tseng G.C. (2007). Penalized and weighted K-means for clustering with scattered objects and prior information in high-throughput biological data. Bioinformatics.

[24] Rousseeuw P.J. (1987). Silhouettes: A graphical aid to the interpretation and validation of cluster analysis. J. Comput. Appl. Math..

[25] Benoit K., Matsuo A., Benoit M.K. (2018). R Package: ‘spacyr’. https://cran.r-project.org/web/packages/spacyr/spacyr.pdf.

[26] Honnibal M., Montani I. (2017). spaCy 2: Natural language understanding with Bloom embeddings, convolutional neural networks and incremental parsing. To Appear.

[27] Feinerer I. (2013). Introduction to the tm Package Text Mining in R. https://cran.r-project.org/web/packages/tm/tm.pdf.

[28] Rinker T. (2018). R Package: ‘textstem’. https://cran.r-project.org/web/packages/textstem/textstem.pdf.

[29] Word Frequency Data (2017). Top 5000 common English Words. http://www.wordfrequency.info.

[30] Feinerer I., Hornik K., Wallace M., Hornik M.K. (2017). Package ‘wordnet’. https://cran.r-project.org/web/packages/wordnet/wordnet.pdf.

[31] Benjamini Y., Hochberg Y. (1995). Controlling the false discovery rate: A practical and powerful approach to multiple testing. J. R. Stat. Soc. Ser. B (Methodol.).

[32] Arion D., Huo Z., Enwright J.F., Corradi J.P., Tseng G., Lewis D.A. (2017). Transcriptome alterations in prefrontal pyramidal cells distinguish schizophrenia from bipolar and major depressive disorders. Biol. Psychiatry.

[33] Bousman C.A., Chana G., Glatt S.J., Chandler S.D., Lucero G.R., Tatro E., May T., Lohr J.B., Kremen W.S., Tsuang M.T. (2010). Preliminary evidence of ubiquitin proteasome system dysregulation in schizophrenia and bipolar disorder: Convergent pathway analysis findings from two independent samples. Am. J. Med. Genet. Part Neuropsychiatr. Genet..

[34] Arion D., Corradi J.P., Tang S., Datta D., Boothe F., He A., Cacace A.M., Zaczek R., Albright C.F., Tseng G. (2015). Distinctive transcriptome alterations of prefrontal pyramidal neurons in schizophrenia and schizoaffective disorder. Mol. Psychiatry.

[35] Sheng Z.H., Cai Q. (2012). Mitochondrial transport in neurons: Impact on synaptic homeostasis and neurodegeneration. Nat. Rev. Neurosci..

[36] Darby M., Yolken R.H., Sabunciyan S. (2016). Consistently altered expression of gene sets in postmortem brains of individuals with major psychiatric disorders. Transl. Psychiatry.

[37] Gandal M.J., Haney J.R., Parikshak N.N., Leppa V., Ramaswami G., Hartl C., Schork A.J., Appadurai V., Buil A., Werge T.M. (2018). Shared molecular neuropathology across major psychiatric disorders parallels polygenic overlap. Science.

[38] Lanz T.A., Reinhart V., Sheehan M.J., Rizzo S.J.S., Bove S.E., James L.C., Volfson D., Lewis D.A., Kleiman R.J. (2019). Postmortem transcriptional profiling reveals widespread increase in inflammation in schizophrenia: A comparison of prefrontal cortex, striatum, and hippocampus among matched tetrads of controls with subjects diagnosed with schizophrenia, bipolar or major depressive disorder. Transl. Psychiatry.

[39] Ramaker R.C., Bowling K.M., Lasseigne B.N., Hagenauer M.H., Hardigan A.A., Davis N.S., Gertz J., Cartagena P.M., Walsh D.M., Vawter M.P. (2017). Post-mortem molecular profiling of three psychiatric disorders. Genome Med..

[40] McGrath L.M., Cornelis M.C., Lee P.H., Robinson E.B., Duncan L.E., Barnett J.H., Huang J., Gerber G., Sklar P., Sullivan P. (2013). Genetic predictors of risk and resilience in psychiatric disorders: A cross-disorder genome-wide association study of functional impairment in major depressive disorder, bipolar disorder, and schizophrenia. Am. J. Med Genet. Part Neuropsychiatr. Genet..

[41] Subramanian A., Tamayo P., Mootha V.K., Mukherjee S., Ebert B.L., Gillette M.A., Paulovich A., Pomeroy S.L., Golub T.R., Lander E.S. (2005). Gene set enrichment analysis: A knowledge-based approach for interpreting genome-wide expression profiles. Proc. Natl. Acad. Sci. USA.

